# Black Cataract

**Published:** 1843-10

**Authors:** 


*Black Cataract.*—At the Academy of Sciences, Paris, May 29, 1843, Magne forwarded the following case of this rare and curious disease:—

A female, above sixty years of aage, had labored under some affection of the eyes, for which she had consulted a great number of oculists. She was quite blind; the eyeballs were prominent; the sclerotica appeared to be thin; the iris well-shaped, but perfectly immoveable; bottom of the pupil dark, as in the healthy state.

From these and other symptoms, the disease was supposed to be amaurosis; but a second examination of the patient was made in a darkened chamber, and with the aid of a candle, as recommended by Sanson. The deep-seated images were absent, and the author accordingly declared the case to be one of black cataract, with adhesion of the iris. The diagnosis having been confirmed by Cruveilhier, the lens on the right side was depressed, on the 25th of March, 1843. The adhesions of the iris were numerous; but as soon as the capsule was lacerated, the dark colour of the lens became evident, and, on depressing it, several black fragments were detached.

On the second day after the operation the pupil appeared to be less contracted, the base being quite dark, but on the following day it was closed by a white substance. Cruveilhier regarded this as the lens, which had come forwards, after having lost its dark colour in the vitreous humor. The operation was unsuccessful, and was, therefore, repeated in a fortnight; but the first touch of the needle showed that the body supposed to be the lens was, in reality, the capsule, which was extremely soft and elastic. A few shreds were removed with much difficulty, and the patient recovered but a very imperfect power of vision.—*Prov. Med. Journ.—Med. Examiner.*

